# Repeatability and reproducibility of post-mortem central corneal thickness measurements using a portable optical coherence tomography system in humans: a prospective multicenter study

**DOI:** 10.1038/s41598-020-71546-1

**Published:** 2020-09-02

**Authors:** Pietro Emanuele Napoli, Matteo Nioi, Letizia Gabiati, Michela Laurenzo, Fabio De-Giorgio, Vincenzo Scorcia, Simone Grassi, Ernesto d’Aloja, Maurizio Fossarello

**Affiliations:** 1 Department of Surgical Science, Eye Clinic, University of Cagliari, Via Ospedale 46, 09124 Cagliari, Italy; 2 Department of Clinical Sciences and Public Health, Forensic Medicine Unit, University of Cagliari, 09124 Cagliari, Italy; 3 Department of Health Surveillance and Bioethics, Section of Legal Medicine, Università Cattolica del Sacro Cuore, Rome, Italy; 4 Fondazione Policlinico Universitario A. Gemelli, IRCCS, Rome, Italy; 5 Medical and Surgical Sciences Department, Ophthalmology Operative Unit, Magna Græcia University, Catanzaro, Italy; 6 Clinica Oculistica, San Giovanni di Dio Hospital, Azienda Ospedaliera Universitaria di Cagliari, 09124 Cagliari, Italy

**Keywords:** Biotechnology, Imaging

## Abstract

To assess the repeatability and reproducibility of post-mortem central corneal thickness (CCT) measurements made by the portable iVue spectra-domain (SD) optical coherence tomography OCT (Optovue Inc, Fremont, CA) system in humans, and to prospectively establish the time-course of CCT after death. In a prospective multicenter setting, CCT measurements were obtained from 58 human eyes at the following 16 time-points after death: *immediately* (within 2 h), and at *each hour* by the next 17 h. The range of CCT values for each subject was determined and longitudinal data were used to illustrate the variation in *open* and *close* eye mode. All measurements were made by two independent and well-trained examiners for session. Main outcome measures were intraclass correlation coefficients (ICC), repeatability and reproducibility coefficients, and coefficients of variation of the average central (0–2 mm). Overall, a total of 5,568 OCT measurements were performed by examiners. The repeatability coefficient varied from 0.3 to 1.7% and the reproducibility coefficient varied from 0.3 to 1.6% throughout the entire experimental time frame. Furthermore, the values of the different ICCs were also high during the different postmortem intervals, thus demonstrating the excellent repeatability and reproducibility of the present OCT approach. When CCT measurements were analyzed longitudinally, corneal thickness showed different behavior based on the *open* or *close* eye mode. The present study demonstrates that portable OCT imaging can be reliably used for corneal pachymetric measurements in supine subjects and during the post mortem period, i.e. without visual fixation and normal physiology/architecture of examined tissues.

## Introduction

Optical coherence tomography (OCT) technology has proven to be a significant diagnostic tool for improving scientific knowledge regarding in vivo structures of the anterior and posterior segment of the eye^1–4^. The use of anterior segment OCT, which provides in vivo imaging of the ocular surface and anterior segment from front to back (the tear film, conjunctiva, individual corneal layers, sclera, angle and lenticular structures), allows to evaluate tissue anatomy at histological resolution and evaluate differences in cellular morphology and patterns, including central corneal thickness (CCT). All these applications help clinicians to diagnose various anterior segment pathologies otherwise not visualized by traditional methods, and to monitor the success and complications of several anterior segment surgical procedures.

Nevertheless, the potential of this instrument to improve the evaluation of the cornea in the postmortem period, e.g. before explant (by means of a quantitative analysis of human CCT and morphological analysis on all planes of the tissue), or to help forensic pathologists in the difficult task to estimate the postmortem interval (PMI) has never been evaluated^5^. Of note, post-mortem corneal evaluation differs from in vivo evaluation due to the lack of visual fixation and to the less than ideal tissue transparency.

Therefore, the aim of our work was to establish the reproducibility and repeatability of a portable OCT system in measuring CCT at different PMIs in human corpses, and to quantify the temporal range of reliability of the method.

## Methods

In this multicenter, prospective and observational study, we evaluated the corneas of human corpses stored in the morgues of two university teaching hospitals, namely the “Fondazione Policlinico Gemelli”, Catholic University of Rome, and the “Policlinico di Monserrato”, University of Cagliari, utilizing a portable spectral-domain OCT (SD-OCT) device (iVue SD-OCT, Optovue Inc, Fremont, CA).

Regular practice was applied to transfer bodies immediately after death to a room with humidity (within a range of 50%–60%) and temperature (within a range of 12–22 °C) monitored over time. In all cases, the *exact* moment of the death was *well known* and easily extrapolated by the medical record as with other clinical data. In no cases eye drops were used to preserve the ocular surface integrity and corneal transparency. Overall, 46 eyes (23 corpses) were studied in Rome and 12 eyes (six corpses) were studied in Cagliari.

Corpses were scrupulously examined before being included in the study, and considered eligible only if associated with unremarkable medical history (collected from review of past medical records), and a normal ophthalmologic examination. Therefore, subjects with external ocular diseases, any evidence of lid abnormality, history of corneal surgery, previous topical or systemic medication, were excluded in both centers.

The present work was conducted ethically according to the principles of the Declaration of Helsinki. Ethical evaluation from local Independent Ethical Committees of the University of Cagliari was achieved for the protocol of the trial, and due to the observational, ‘non-pharmacological’, and non-invasive nature of the procedure a formal approval was not considered due (*Regolamento di Polizia mortuaria,* DPR 285/90). Informed consents of next of kin were obtained by appropriate district attorney. To note, all the corpses were under criminal investigation by the in charge Prosecutor at the local Judicial District and the authorization to perform the non-intrusive measurements was directly released to the physicians who eventually carried out the autopsy.

### Portable OCT platform

The iVue SD-OCT is a commercially available device working at a 5-µm axial resolution and an image acquisition rate of 26,000 axial scans per second, with a frame rate of 256 to 1,024 A-scan/frame. This SD imaging machine uses a center wavelength of 840 ± 10 nm to provide high-resolution scans. All human corneas were cross-sectionally studied by the pachymetry mapping protocol. The latter consists of eight radial lines composed by 1,020 A-scans, with 22.5° of interval and 6 mm of length. Two examiners in Rome (L.G., M.L.) and in Cagliari (P.N., M.N.), named operator n.1, n.2, n.3 and n.4, respectively, scanned all corneas. The iVue software (Optovue, Fremont, California, USA) defined automatically all measurements. For the aims of the present paper, repeatability and reproducibility analysis was focus on *central* corneal thickness (0–2 mm).

### Experimental setting and OCT imaging

At each OCT scanning session, all human cadavers were maintained in supine position and corneal reflex was monitored and sought to obtain high quality images of the central cornea. In all subjects, the right eye was held in close mode (by a mechanical support) and the left eye in open mode.

Overall, OCT imaging was performed at the following 16 time-points after death: *immediately* (within 2 h), and at *each hour* by the next 17 h. Following this scanning protocol, human corneas underwent OCT imaging with the pachymetry mapping protocol. Specifically, three scans were performed by examiner n.1 or n.3 and further three scans by operator n.2 or n.4 in the shortest possible interval.

The real-time video image (on PC) was used to align the corneal vertex to the centre of the OCT image. Moreover, the good quality of exams was considered as a necessary condition to guarantee sufficient reliability of the method. A good demarcation of the corneal boundaries and the absence of artifacts, were all criteria to evaluate the image quality. Accordingly, images were excluded whenever poorly focused, not centered, or low (less than 30) in scan score index (SSI).

### Statistical analysis

All statistical analyses were performed by Statistical Package for Social Science SPSS version 21.0 (SPSS Inc., Chicago, Illinois). For each OCT session, results were summarized as Mean and SD of average CCT measurements (0–2 mm). The Lilliefors test and Shapiro–Wilk test were carried out for normality.

For reliability assessment were determined reproducibility and repeatability coefficients, and intraclass correlation coefficients (ICCs). The latter were calculated from the variance for mixed models for each state as proposed by Carpenter and Bartko^6^. Thus, the values close to 1 indicated high reproducibility/repeatability.

As suggested by the British Standard Institution and indicated by Bland and Altman, the reproducibility coefficients were calculated as 2 SDs of the differences between measurements performed by different observers during repetition of OCT scans at the same PMI, divided by the average of the means of each pair of readings^7–9^. The repeatability coefficient was determined dividing 2 SDs of the differences between pairs of reads at the same PMI in the same ocular globe, with SDs of the differences between pairs of reads at the same PMI in the same eye by the same observer.

For each PMI session, statistically significant differences (intra-observer and/or inter-observer) between measurements were evaluated by the Wilcoxon matched-pairs test (5% significance level).

Approximately six individual measurements are needed before a correlation coefficient of 0.930 is said to be statistically significant, with an accepted alpha risk of 0.05 and a β risk of 0.20 (i.e., 80% statistical power) in a two-sided test.

Percentage changes in corneal thickness (ΔCCT%) were calculated as follows:

$$\frac{{\left( {CCT2 - CCT1} \right)}}{CCT1} \times 100$$

where *CCT*_2_—final CCT (i.e., measurement at the 17th hour), and *CCT*_1_—baseline CCT. Relationship between ΔCCT% and demographics or baseline CCT was determined by calculating Pearson χ square test and Spearman’s ρ test / Kendall’s τ test.

*p* values less than 0.05 were considered significant.

## Results

Overall, we studied 58 eyes of 29 subjects (69.73 ± 19.65 years [mean ± SD], 48.27% female). During the OCT imaging section, no changes in corneal transparency were observed in the first 17 h.

The mean and SDs of CCT measurements in the central region (0–2 mm) obtained at each scanning session are presented in Tables 1 and 2. A total of 4,416 and 1,152 measurements were performed, respectively, in Rome and in Cagliari.

**Table 1:** Average corneal thickness measurements (obtained in Rome).

PMI	Close mode	Open mode
	Mean ± SD	Mean ± SD
2nd hour	659.1 ± 12.4	629.0 ± 10.2
3rd hour	660.3 ± 13.5	619.0 ± 8.0
4th hour	680.0 ± 22.0	453.1 ± 14.5
5th hour	618.5 ± 40.1	535.9 ± 45.2
6th hour	652.1 ± 45.2	471.3 ± 43.1
7th hour	638.1 ± 40.2	404.0 ± 42.5
8th hour	667.3 ± 46.3	393.3 ± 42.3
9th hour	677.3 ± 41.5	389.0 ± 42.1
10th hour	704.9 ± 43.3	375.8 ± 43.1
11th hour	697.2 ± 42.6	372.2 ± 42.1
12th hour	730.3 ± 45.6	373.1 ± 40.4
13th hour	726.8 ± 44.8	366.5 ± 40.6
14th hour	793.4 ± 44.3	392.2 ± 40.2
15th hour	765.3 ± 43.3	369.2 ± 39.5
16th hour	978.5 ± 28.4	349.5 ± 39.6
17th hour	999.8 ± 28.4	348.6 ± 39.2

Data are expressed at the mean ± SD (in μm).

**Table 2:** Average corneal thickness measurements (obtained in Cagliari).

PMI	Close mode	Open mode
	Mean ± SD	Mean ± SD
2nd hour	601.3 ± 12.1	600.3 ± 10.7
3rd hour	605.4 ± 12.7	598.0 ± 7.6
4th hour	625.0 ± 24.1	456.2 ± 12.5
5th hour	611.4 ± 30.1	530.3 ± 41.2
6th hour	645.3 ± 41.2	465.5 ± 41.2
7th hour	618.8 ± 36.2	401.0 ± 40.5
8th hour	642.6 ± 41.3	387.5 ± 38.7
9th hour	651.4 ± 38.2	381.2 ± 42.1
10th hour	676.4 ± 41.5	366.1 ± 41.1
11th hour	672.7 ± 40.9	361.7 ± 39.1
12th hour	700.1 ± 42.2	369.4 ± 40.4
13th hour	698.3 ± 41.6	361.7 ± 37.8
14th hour	703.7 ± 42.3	363.1 ± 40.2
15th hour	671.5 ± 39.3	341.5 ± 36.5
16th hour	878.5 ± 24.2	323.4 ± 35.6
17th hour	945.3 ± 27.2	348.6 ± 39.2

Data are expressed at the mean ± SD (in micrometers).

The coefficients of repeatability and reproducibility, and the coefficients of variation are reported in Tables 3 and 4. These data should be evaluated taking into account the average values of the average CCT (Tables 1, 2) and the axial resolution of OCT system.

**Table 3:** Intraclass correlations and coefficients of repeatability and reproducibility (obtained in Rome).

PMI	Close mode							Open mode						
	ICC_(1)_ (intra)	ICC_(2)_ (intra)	ICC_(1–2)_ (inter)	CoR %	CV_w_%	CoR_1–2_%	CV_w_%	ICC_(1)_ (intra)	ICC_(2)_ (intra)	ICC_(1–2)_ (inter)	CoR %	CV_w_ %	CoR_1–2_%	CV_w_ %
2nd	0.985	0.982	0.966	0.5 (0.4–0.6)	0.2	0.4 (0.3–0.5)	0.2	0.984	0.983	0.965	0.4 (0.3–0.6)	0.3	0.3 (0.4–0.5)	0.2
3rd	0.956	0.952	0.937	0.6 (0.5–0.7)	0.3	0.5 (0.4–0.7)	0.4	0.955	0.954	0.936	0.5 (0.4–0.7)	0.3	0.4 (0.3–0.7)	0.3
4th	0.945	0.947	0.938	0.5 (0.4–0.7)	0.2	0.4 (0.3–0.7)	0.2	0.946	0.946	0.937	0.6 (0.4–0.7)	0.2	0.3 (0.3–0.6)	0.2
5th	0.936	0.935	0.935	1.5 (1.3–1.6)	1.1	1.4 (1.3–1.5)	1.2	0.934	0.933	0.934	1.4 (1.3–1.6)	1.2	1.3 (1.2–1.5)	1.1
6th	0.935	0.937	0.936	1.6 (1.4–1.8)	1.2	1.3 (1.4–1.5)	1.2	0.936	0.935	0.939	1.5 (1.4–1.7)	1.3	1.2 (1.4–1.5)	1
7th	0.937	0.936	0.942	1.4 (1.2–1.5)	1.1	1.3 (1.1–1.5)	1.2	0.935	0.934	0.941	1.3 (1.2–1.4)	1.2	1.3 (1.1–1.4)	1.2
8th	0.938	0.937	0.935	1.3 (1.1–1.5)	1.0	1.2 (1.1–1.4)	1.0	0.937	0.938	0.942	1.2 (1.1–1.4)	1.1	1.1 (1.1–1.3)	1.0
9th	0.934	0.935	0.955	1.7 (1.5–1.8)	1.4	1.6 (1.4–1.7)	1.5	0.933	0.934	0.934	1.6 (1.5–1.7)	1.5	1.5 (1.4–1.6)	1.3
10th	0.957	0.958	0.937	0.7 (0.4–0.9)	0.9	0.6 (0.3–0.9)	1.3	0.956	0.957	0.936	0.6 (0.4–0.8)	1	0.5 (0.3–0.7)	1.2
11th	0.953	0.952	0.935	0.6 (0.5–0.7)	0.3	0.5 (0.5–0.8)	0.3	0.952	0.951	0.934	0.5 (0.4–0.7)	0.4	0.4 (0.5–0.7)	0.3
12th	0.945	0.946	0.947	0.5 (0.4–0.8)	0.2	0.4 (0.4–0.7)	0.3	0.946	0.945	0.936	0.4 (0.3–0.8)	0.2	0.3 (0.4–0.6)	0.2
13th	0.944	0.943	0.946	0.4 (0.2–0.5)	0.2	0.3 (0.2–0.4)	0.2	0.943	0.944	0.935	0.3 (0.1–0.5)	0.3	0.3 (0.2–0.4)	0.1
14th	0.945	0.946	0.947	0.5 (0.3–0.7)	0.2	0.4 (0.2–0.7)	0.3	0.946	0.945	0.936	0.4 (0.3–0.6)	0.2	0.3 (0.2–0.6)	0.3
15th	0.952	0.951	0.935	0.6 (0.4–0.7)	0.3	0.5 (0.4–0.6)	0.3	0.953	0.952	0.934	0.5 (0.3–0.7)	0.4	0.4 (0.3–0.6)	0.4
16th	0.951	0.953	0.934	0.6 (0.4–0.8)	0.3	0.5 (0.3–0.8)	0.4	0.952	0.953	0.933	0.5 (0.4–0.7)	0.3	0.5 (0.3–0.8)	0.5
17th	0.954	0.952	0.936	0.5 (0.3–0.6)	0.2	0.4 (0.2–0.6)	0.2	0.953	0.952	0.935	0.4 (0.3–0.5)	0.3	0.3 (0.1–0.5)	0.2

ICC, Type C intraclass correlation coefficients using a consistency definition: the between-measure variance is excluded from the denominator variance. The estimator is the same, whether the interaction effect is present or not. Data in parentheses represent 95% CI. Number 1 represents measurements by observer 1. Number 2 represents repeat measurements by observer 2 at the same session (time-point). Number 1–2 represents repeat measurements by observer 1 and 2 at the same session (time-point).

CoR, coefficient of repeatability; CoR1–2, coefficients of reproducibility; CVw, coefficient of variation. CoR and CVw are expressed as percentages (Coefficients % = Coefficients × 100).

**Table 4:** Intraclass correlations and coefficients of repeatability and reproducibility (obtained in Cagliari).

PMI	Close mode							Open mode						
	ICC_(3)_ (intra)	ICC_(4)_ (intra)	ICC_(3–4)_ (inter)	CoR %	CV_w_%	CoR_3–4_%	CV_w_%	ICC_(3)_ (intra)	ICC_(4)_ (intra)	ICC_(3–4)_ (inter)	CoR %	CV_w_ %	CoR_3–4_%	CV_w_ %
2nd	0.995	0.983	0.967	0.6 (0.4–0.7)	0.1	0.5 (0.3–0.5)	0.2	0.985	0.984	0.985	0.5 (0.3–0.6)	0.2	0.4 (0.3–0.5)	0.2
3rd	0.966	0.954	0.938	0.7 (0.5–0.8)	0.3	0.6 (0.4–0.7)	0.3	0.955	0.954	0.946	0.5 (0.4–0.7)	0.2	0.4 (0.3–0.7)	0.2
4th	0.956	0.948	0.939	0.4 (0.3–0.7)	0.2	0.5 (0.3–0.7)	0.1	0.947	0.948	0.947	0.7 (0.4–0.8)	0.1	0.4 (0.3–0.6)	0.2
5th	0.946	0.938	0.936	1.4 (1.3–1.6)	1.0	1.4 (1.3–1.5)	1.1	0.936	0.934	0.940	1.5 (1.3–1.6)	1.1	1.4 (1.2–1.5)	1.0
6th	0.946	0.938	0.939	1.7 (1.4–1.8)	1.1	1.4 (1.3–1.5)	1.0	0.937	0.936	0.939	1.6 (1.4–1.7)	1.0	1.3 (1.4–1.5)	0.8
7th	0.947	0.938	0.943	1.5 (1.2–1.6)	0.9	1.3 (1.1–1.5)	1.1	0.941	0.938	0.941	1.4 (1.2–1.5)	1.1	1.3 (1.1–1.4)	1.1
8th	0.948	0.939	0.937	1.2 (1.1–1.5)	1.0	1.3 (1.1–1.4)	0.9	0.939	0.938	0.942	1.3 (1.1–1.4)	0.9	1.2 (1.1–1.3)	0.8
9th	0.935	0.935	0.956	1.6 (1.5–1.8)	1.3	1.7 (1.4–1.8)	1.3	0.938	0.937	0.934	1.6 (1.5–1.7)	1.3	1.5 (1.4–1.6)	1.2
10th	0.958	0.959	0.938	0.9 (0.4–0.9)	0.8	0.7 (0.3–0.9)	1.2	0.957	0.956	0.952	0.7 (0.4–0.8)	1	0.6 (0.3–0.7)	1.1
11th	0.963	0.952	0.936	0.6 (0.5–0.7)	0.2	0.6 (0.5–0.8)	0.2	0.953	0.955	0.934	0.6 (0.4–0.7)	0.3	0.4 (0.5–0.7)	0.3
12th	0.955	0.947	0.946	0.8 (0.4–0.9)	0.1	0.5 (0.4–0.7)	0.2	0.948	0.945	0.936	0.5 (0.3–0.8)	0.2	0.4 (0.3–0.6)	0.2
13th	0.945	0.943	0.945	0.6 (0.2–0.5)	0.2	0.5 (0.2–0.4)	0.1	0.945	0.944	0.936	0.4 (0.1–0.5)	0.3	0.3 (0.2–0.6)	0.1
14th	0.946	0.947	0.946	0.5 (0.3–0.7)	0.3	0.5 (0.2–0.7)	0.3	0.947	0.946	0.936	0.5 (0.3–0.6)	0.1	0.3 (0.2–0.7)	0.3
15th	0.956	0.953	0.935	0.7 (0.4–0.8)	0.4	0.5 (0.4–0.6)	0.4	0.955	0.953	0.935	0.5 (0.3–0.7)	0.3	0.4 (0.3–0.8)	0.5
16th	0.960	0.954	0.936	0.6 (0.4–0.8)	0.3	0.6 (0.3–0.8)	0.5	0.954	0.954	0.934	0.5 (0.4–0.7)	0.3	0.4 (0.3–0.8)	0.6
17th	0.956	0.953	0.937	0.6 (0.3–0.7)	0.1	0.4 (0.2–0.6)	0.2	0.956	0.954	0.938	0.5 (0.3–0.6)	0.2	0.3 (0.1–0.5)	0.3

ICC, Type C intraclass correlation coefficients using a consistency definition: the between-measure variance is excluded from the denominator variance. The estimator is the same, whether the interaction effect is present or not. Data in parentheses represent 95% CI. Number 3 represents measurements by observer 3. Number 4 represents repeat measurements by observer 4 at the same session (time-point). Number 3–4 represents repeat measurements by observer 3 and 4 at the same session (time-point).

CoR, coefficient of repeatability; CoR3–4, coefficients of reproducibility; CVw, coefficient of variation. CoR and CVw are expressed as percentages (Coefficients % = Coefficients × 100).

The ICC values for intra-observer, inter-observer and inter-session reproducibility are also shown in Tables 3 and 4.

**Figure 1:**
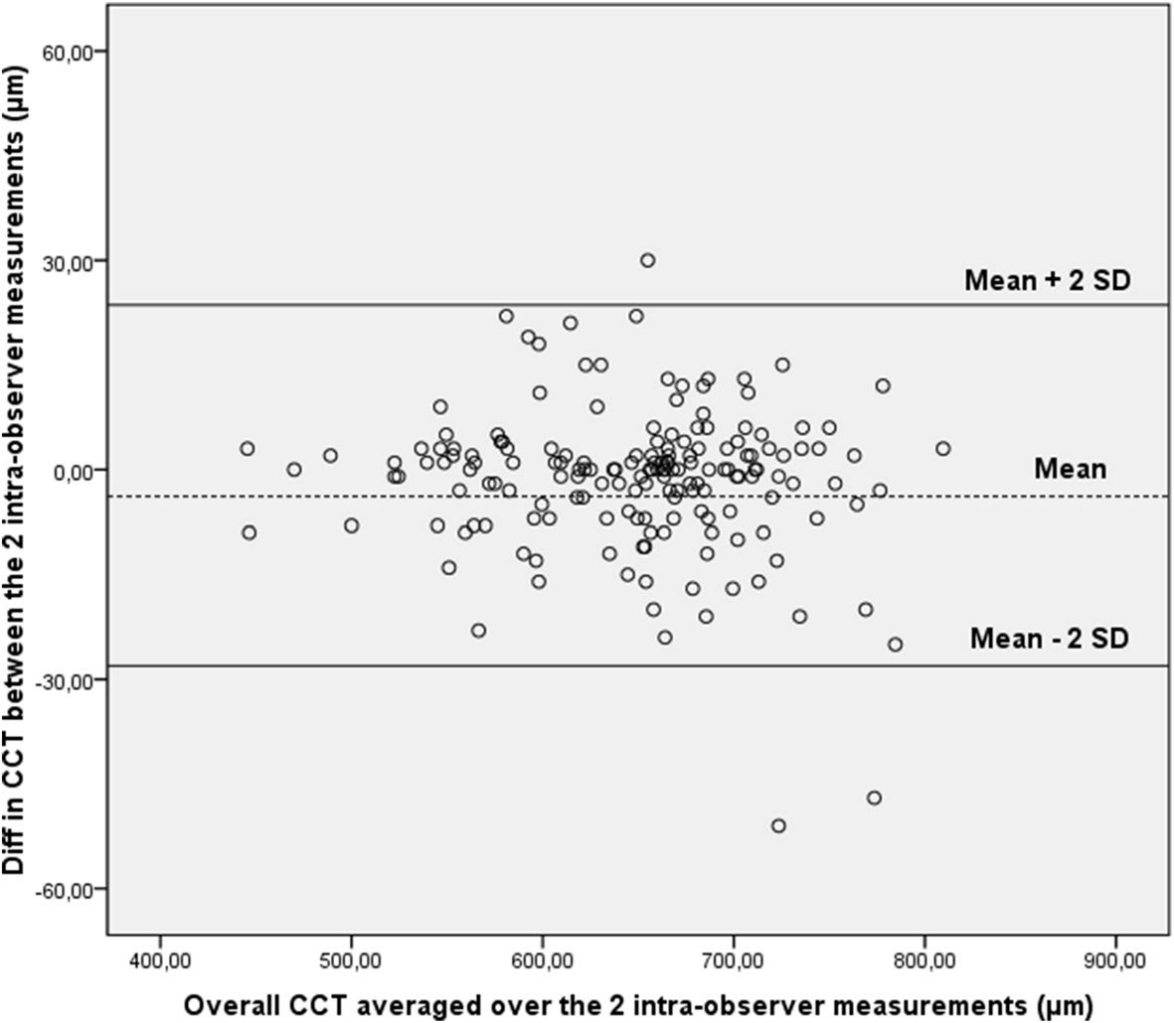
Bland–Altman plot of repeatability of central corneal thickness (CCT) measurements. Mean central corneal thickness (CCT) for paired intra-observer measurements is plotted against the difference in CCT between the two results. Overall, 95% of the values fell within 2 SDs of the mean.

**Figure 2:**
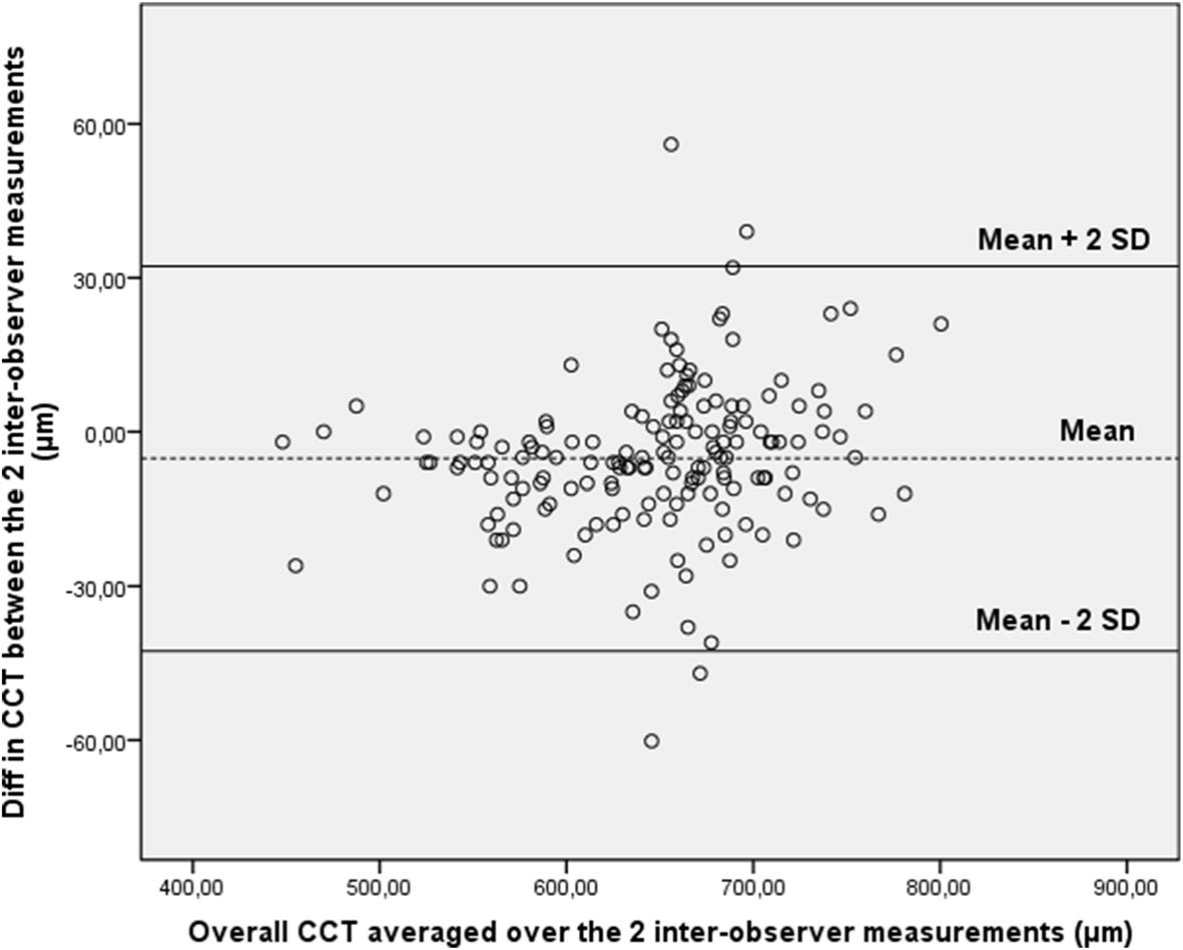
Bland–Altman plot of reproducibility of central corneal thickness (CCT) measurements obtained by the first observers (n.1 and n.3) and the second observers (n.2 and n.4). Mean central corneal thickness (CCT) for paired inter-observer measurements (obtained by the first operators and the second operators) is plotted against difference in CCT between the two results. Ninety-five percent of the values fell within 2 SDs of the mean.

Differences against means were plotted in graphics for the CCT measurements made by operators (Figs. 1 and 2). In all cases, ≥ 95% of the differences were found to fall within 2 SD of the mean. These results indicate repeatability in post mortem CCT measurements according to the definitions of the British Standards Institution.

Overall, no significant association was found between the within-subject SD and mean inter-observer, intra-observer, or intra-session CCT measurements. Similarly, no significant systematic differences were revealed between OCT measurements obtained by different scan sessions using the Wilcoxon matched-pairs test.

**Figure 3:**
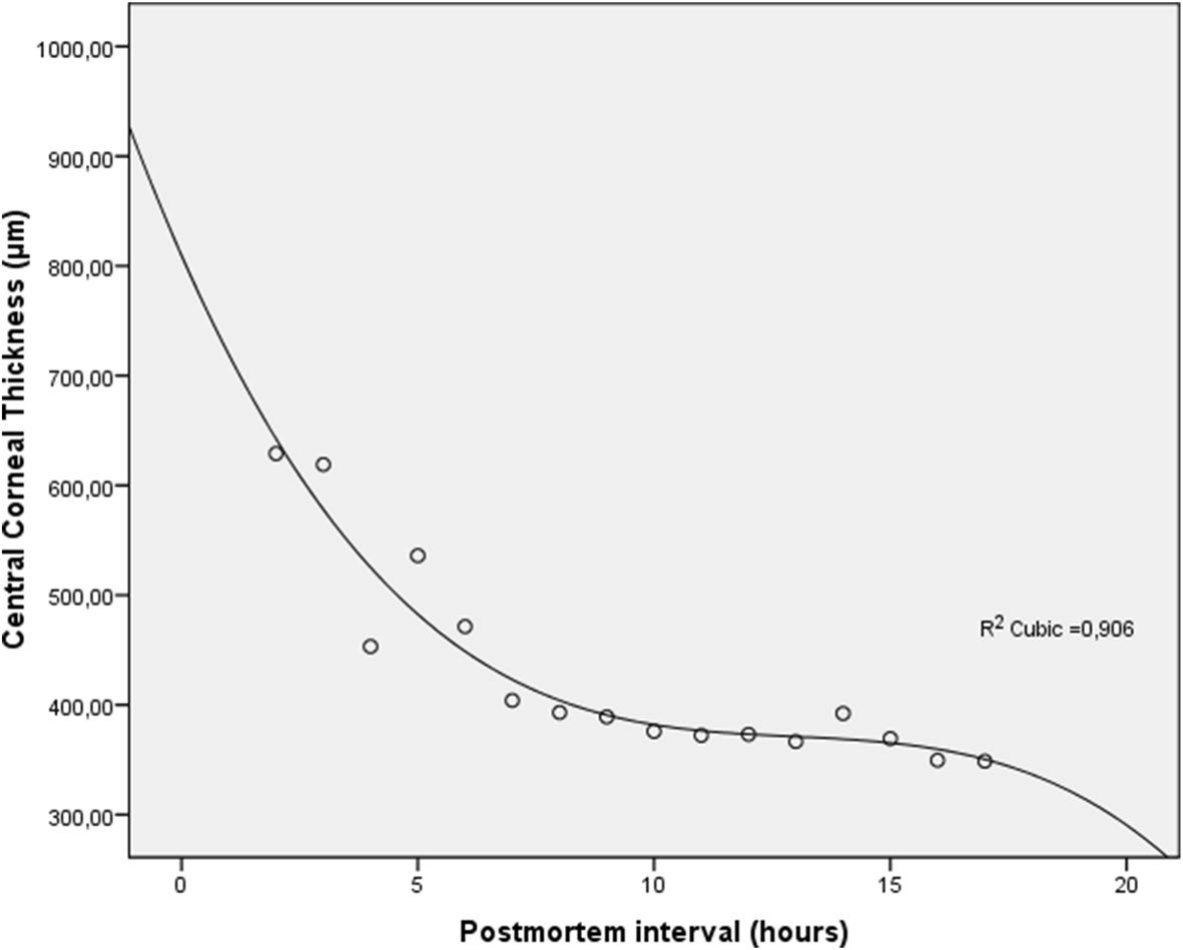
Central corneal thickness (CCT) measured by optical coherence tomography versus postmortem intervals in *open* mode. CCT decreases progressively after death describing an S-shaped model (R^2^ cubic function).

**Figure 4:**
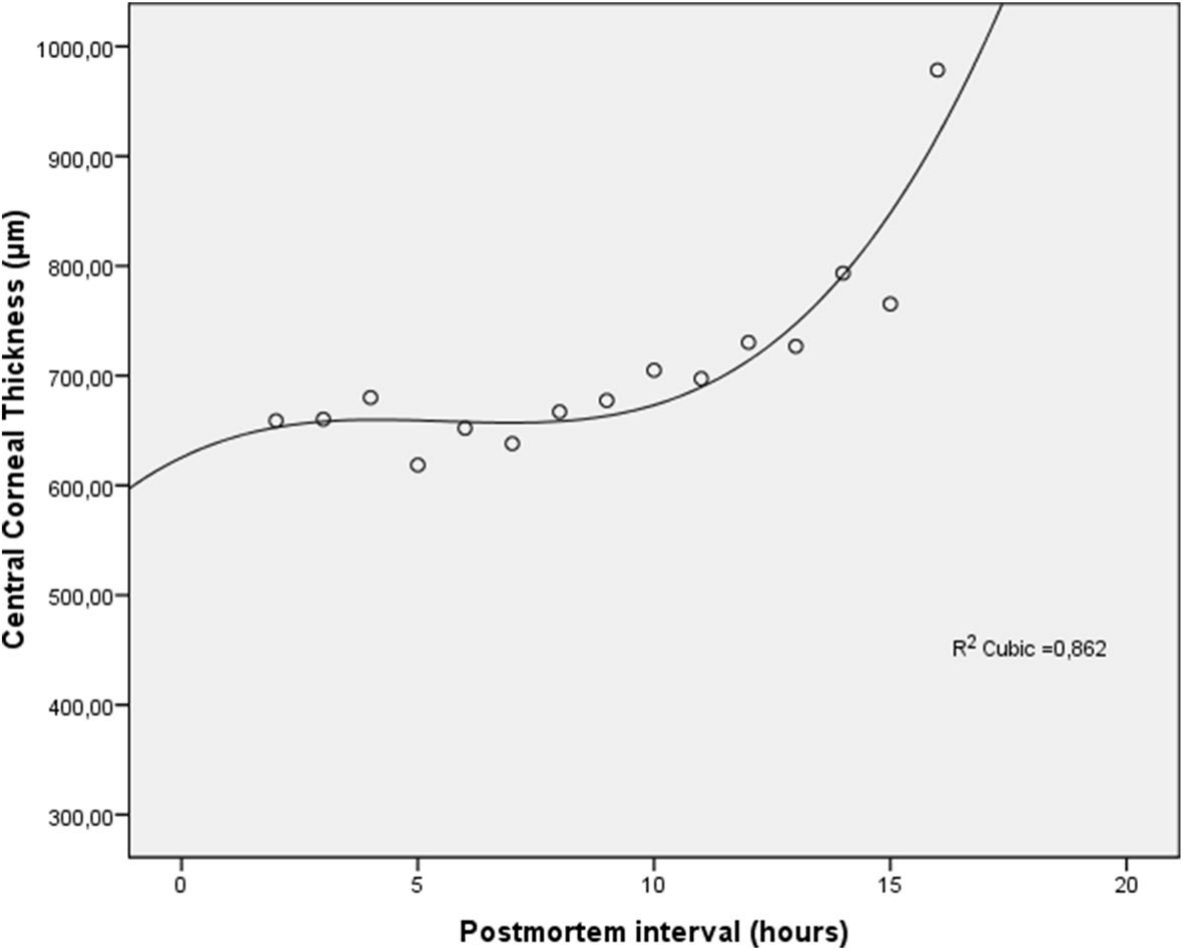
Central corneal thickness (CCT) measured by optical coherence tomography versus postmortem intervals in *close* mode. CCT increases progressively after death describing an S-shaped model (R^2^ cubic function).

Corneal thickness changes are reported in Figs. 3 and 4. As can be seen, corneal thickness showed opposite behaviors based on the *open* or *close* eye mode. Accordingly, ΔCCT% showed two different distributions in relation to the eye opening setting: 16.918 ± 15.580 in *close* mode, and − 21.495 ± 16.202 in *open* mode.

Correlation analysis unveiled in *open* eye mode a negative relationship between ΔCCT% and baseline CCT (Spearman’s ρ was − 0.710, *p* < 0.001; Kendall’s τ was − 0.509, *p* < 0.001), and in *close* eye mode a mild degree of relationship between the same two variables (Spearman’s ρ was − 0.458, *p* = 0.018; Kendall’s τ was − 0.311, *p* = 0.026). No significant association was found between ΔCCT% and demographic characteristics (i.e. gender or age).

## Discussion

Although OCT pachymetric measurements have been described after death on animal model, this article reports for the first time the reliability and longitudinal measurements of OCT corneal pachymetry in human cadavers^5^. This postmortem imaging approach may be helpful in studying donors’ corneas before explant for corneal transplantation, and it can allow forensic pathologists to better understand, and to record in an objective manner, corneal changes in corpses for a quantitative assessment of human forensic PMIs.

Most of the classic pachymetric techniques (e.g. ultrasound probe sonography) offer only *point* measurements and/or a *contact* approach (potentially deforming the corneal structure, especially in an ongoing context as the postmortem reduction of intraocular pressure). Moreover, the accurate probe alignment by the operator is also necessary for reliable measurements (examiner dependence). Other pachymetric methods can be used only in the sitting position (e.g. rotating Scheimpflug camera). Overall, these *traditional* methods have proven to be reliable, but they do not offer the possibility of combining morphological analysis of the corneal layers with quantitative analysis^10–17^.

Conversely, portable OCT system can overcome all these limitations. Its characteristics of easy handling and portability on the site where the corpse lies, the ability to record high-resolution scans for monitoring changes, the ability to acquire in a fast and non-contact mode, make OCT imaging an ideal tool in the field of corneal transplantology and forensic sciences. In fact, the present study demonstrates that this high-technology instrument can be reliably used for corneal pachymetric measurements in supine subjects and during the post mortem period, i.e. without visual fixation and normal physiology/architecture of the examined tissue. The repeatability coefficient varied from 0.3 to 1.7% and the reproducibility coefficient varied from 0.3 to 1.6% throughout the overall experiment time span. Furthermore, the values of the different ICCs were also high during the different PMIs, thus demonstrating the high repeatability and reproducibility of the present OCT approach.

With iVue SD-OCT, the intra- and inter-operator repeatability of post mortem CCT measurements is promoted by an algorithm that identifies the signal peaks of the posterior and anterior corneal borders, and by the high speed of image acquisition.

Several features of OCT imaging that we demonstrated are very desirable for pachymetric monitoring in the postmortem period. Firstly, the high-speed OCT system reliably acquires CCT data in a short time (generally faster than ultrasound technologies) to reduce time-related artifacts. Secondly, the high definition of OCT images allows operators to clearly recognize the margins of the corneal tissue and the precise corneal vertex landmark at all time-points. Unlike ultrasound or other methods, OCT measurement does not require contact or immersion (gel products imply an alteration of the corneal metabolism and structure), nor precise basic patient position.

It is interesting to note that, in evaluation of human cadavers, the tendency for OCT to overestimate CCT measurements (by adding human tear thickness) with respect to ultrasound (i.e. the *gold standard*) is implicitly reduced or absent (*methodological bias*). In fact, both techniques do not include in this case the lacrimal thicknesses (ultrasound probe always moves it). In other words, the tear film evaporates after death and it is consequently difficult to detect also for OCT. Therefore, the absence of significant CCT variations due to reflex tearing may be a reason for the high repeatability of results. Furthermore, in comparison with point measurements, the average pachymetry on the central 2 mm tends to limit measurement errors.

Based on our data, pachymetry measurements were highly reproducible in all detection times (up to the 17th hour). Since the anterior and posterior boundary peaks were less identifiable in later PMIs, we believe these measurements might become less and less accurate in a longer time period.

When CCT measurements were performed longitudinally in human cadavers, a reduction in corneal thickness was observed in the *open* eye mode. This decrease in the corneal thickness is probably related to the progressive *dehydration* of the tissue, which is in turn favored by the continuous tear film evaporation without any replacement naturally occurring after death^18^. Accordingly, a rapid reduction in CCT was detected by OCT in the early PMIs (Fig. 3). Subsequently, a steady state of the corneal thickness was observed, presumably due to the balanced replacement of the liquid lost by evaporation with that coming from the anterior chamber (Fig. 3). Of note, the correlation analysis indicated that thinner corneas thin more after death, thus suggesting that the latter are more susceptible to changes in CCT values under non-physiological conditions.

On the contrary, CCT values progressively increased in all *close* eye mode corneas during the postmortem period, in which evaporation contributed to a lesser extent to the thinning of the tear film, but the liquid moving under osmotic pressure from the anterior chamber (i.e. aqueous humor) accumulated in the various corneal layers (sharply after the 9th hour) due to a *dysfunction* of the corneal endothelium (Fig. 4)^18^. Moreover, corneal *hypoxia* was also likely to play a role in tissue swelling.

As regards the overall changes of the metric parameters, it is also presumable that the rapid passage of liquids through the cornea in the immediate hours after death may be facilitated by the involvement of the *aquaporins* water channels, located both at the level of the anterior and posterior cornea, in addition to passive diffusion^19^. Moreover, the impairment and final loss of tight junctions function at both side of corneal borders (endothelium and epithelium), with consequent derangement of structural features and progressive failure of corneal shield function, may also have played a crucial role.

In summary, we have demonstrated for the first time the excellent reliability of corneal pachymetry in human cadavers using a portable OCT system, as well as the existence of different behaviors of the related tissues on the basis of eye opening mode. Concluding, it is possible to state that this device should be valuable for corneal explantation or, in general, for corneal transplantology, and for forensic sciences.^20^
